# Novel developments in plant organellar signalling

**DOI:** 10.1093/jxb/erw245

**Published:** 2016-06-20

**Authors:** Markus Teige

**Affiliations:** University of Vienna

Subcellular compartmentation is a hallmark of all eukaryotic cells and in the past has mostly been discussed in terms of a separation of different metabolic pathways. However, in the cellular context these different activities need to be coordinated and adjusted to changing environmental conditions involving a great variety of different signalling mechanisms and pathways. While some of them have been known for many years, a number of completely novel pathways and signalling mechanisms have been discovered only recently. They involve a surprisingly broad range of metabolites, secondary messengers and hormones, as well as proteins such as transcription factors and protein kinases. At the time of the last *Journal of Experimental Botany* special issue on organellar signalling in 2012 (e.g. see Teige, 2012) there was a focus on identification of novel components and description of general mechanisms. Much progress has been achieved since then, particularly in the latter area, and therefore here we emphasize emerging novel connections between different signalling pathways and the specific mechanisms involved.

## Reviews – novel connections and specific mechanisms

Reviews comprise the first part of the special issue, and Kmiecik *et al*. (pages 3793–3807) introduce the subject, briefly summarizing different signalling pathways involved in organellar signalling and highlighting emerging topics, including a group of transcription factors and their potential regulation by phosphorylation or crosstalk with hormones. Opportunities for future research are noted, with a particular focus on chloroplasts. The following review by Wagner *et al.* (pages 3809–3829) summarizes the role of mitochondria in organellar signalling and compares the regulation of mitochondrial Ca^2+^ signalling in plant and animal mitochondria. Both mitochondria and chloroplasts are a potential source of reactive oxygen species (ROS), which are increasingly being identified as important signalling molecules. Their production and role in signalling is described by Mignolet-Spruyt *et al*. (pages 3831–3844), and also addressed in the review by Serrano *et al*. (pages 3845–3854) – they emphasize in particular the emerging role of chloroplasts as central integrators of environmental signals, and their crucial functions during the establishment of plant defence signalling.

Moving from the organelles to signalling molecules and mechanisms, the following reviews focus on the role of protein phosphorylation and kinases. First, the subcellular localization and targets of calcium-dependent protein kinases are covered by Simeunovic *et al*. (pages 3855–3872), with the aim of better understanding the complexity of cellular Ca^2+^ signalling and crosstalk with other kinase pathways based on specific localization of the enzymes. The following survey of protein phosphorylation in chloroplasts by Baginsky (pages 3873–3882) gives an overview of the current status of phosphoproteome analyses in chloroplasts of Arabidopsis, rice and maize, and compares the data in the light of phosphorylation site conservation. This is followed on by Grieco *et al*. (pages 3883–3896), who summarize our current knowledge of thylakoid protein phosphorylation sites involved in the regulation of photosynthesis in plants and green algae from an evolutionary perspective. This approach has uncovered novel phosphorylation hotspots and could help to identify phosphosites with potential functional implications.

The opinion paper by Roustan *et al*. (pages 3897–3907) again extends this approach to gain insights into the evolutionary development of cellular signalling by analysing the highly conserved AMPK-TOR signalling pathway, the key regulator of cellular energy homeostasis in the three domains of life. This cellular signalling module is highly relevant as it involves coordination between energy metabolism and growth processes in different subcellular compartments, with fundamental implications beyond plants.

## Retrograde signalling, light and calcium signalling

In the second part of the special issue research papers address the role of different components of retrograde signalling pathways, light and calcium signalling, as well as protein phosphorylation. In their paper Sun *et al*. (pages 3909–3924) report the identification and characterization of mitochondrial transcription termination factor 4 (mTERF4), which cooperates with GUN1 to regulate plastid gene expression and plastid retrograde signalling. Magnesium chelatase has also been implicated in retrograde signalling in higher plants, and in their analysis of chlorophyll biosynthesis genes Brzezowski *et al*. (pages 3925–3938) show that the Mg chelatase CHLI2 cannot substitute for CHLI1 in chlorophyll synthesis and retrograde signalling in *Chlamydomonas reinhardtii*.

Light-harvesting proteins are a typical target of retrograde signalling pathways, but they also need to react to external stimuli. This becomes particularly relevant in highly variable ocean environments, and this is further described by Taddei *et al*. (pages 3939–3951) who show how multiple stress signalling pathways regulate LHCX family gene expression in the diatom *Phaeodactylum tricornutum* to efficiently attune the acclimation response. Light signals are also frequently employed in developmental processes or physiological acclimation processes in higher plants. Blue light, for example, presents the major stimulus for phototropism or chloroplast positioning in response to changing light conditions. In their study, Łabuz *et al*. (pages 3953–3964) show that the localization of loosely bound calcium in Arabidopsis mesophyll changes under strong blue light in the wild type, but not in *phot2* and *phot1 phot 2* mutants. This indicates that PHOT2 is involved in calcium homeostasis and further supports the close connection between blue light signals and Ca^2+^ signalling.

## Calcium signalling, protein phosphorylation, transcriptional regulation

Ca^2+^ signals in amyloplasts and chloroplasts of Arabidopsis cell suspension cultures are described by Sello *et al*. (pages 3965–3974), who dissect stimulus-specific differential Ca^2+^ responses of non-green plastids versus chloroplasts to several environmental cues using the Ca^2+^ reporter aequorin. Lohscheider *et al*. (pages 3975–3984) show that the core plastoglobule proteome consists of 30 proteins and several transiently associated proteins. Based on careful evaluation of published proteomics data, they conclude that nine of these proteins have one or more serine or threonine phosphorylation sites. Ruge *et al.* (pages 3985–3996) show that the calmodulin-like proteins AtCML4 and AtCML5 are targeted to the endomembrane system by an N-terminal signal-anchor sequence, where they localize as single-pass membrane proteins at the interface between Golgi and the endosomal system. They further speculate that AtCML4 and AtCML5 might provide a basis for calcium regulation of endosomal vesicle transport in higher plants. Although calcium signalling is usually considered to be a typical eukaryotic signalling mechanism, increasing evidence has emerged of an important role in prokaryotes. In this context Walter *et al*. (pages 3997–4008) show that calcium affects the primary cellular metabolism of *Anabaena* under conditions replete in both combined nitrogen and inorganic carbon. They further report that opposite transcriptome responses to calcium treatments occur for nitrogen- and carbon-related processes.
